# The microbiology of impetigo in Indigenous children: associations between *Streptococcus pyogenes*, *Staphylococcus aureus,*scabies, and nasal carriage

**DOI:** 10.1186/s12879-014-0727-5

**Published:** 2014-12-31

**Authors:** Asha C Bowen, Steven YC Tong, Mark D Chatfield, Jonathan R Carapetis

**Affiliations:** Menzies School of Health Research, Charles Darwin University, Darwin, Northern Territory Australia; Princess Margaret Hospital for Children, Perth, Western Australia Australia; Telethon Kids Institute, University of Western Australia, Perth, Western Australia Australia; Royal Darwin Hospital, Darwin, Northern Territory, Australia

**Keywords:** Impetigo, Tropical, Endemic, Staphylococcus aureus, Streptococcus pyogenes, Scabies

## Abstract

**Background:**

Impetigo is caused by both *Streptococcus pyogenes* and *Staphylococcus aureus*; the relative contributions of each have been reported to fluctuate with time and region. While *S. aureus* is reportedly on the increase in most industrialised settings, *S. pyogenes* is still thought to drive impetigo in endemic, tropical regions. However, few studies have utilised high quality microbiological culture methods to confirm this assumption. We report the prevalence and antimicrobial resistance of impetigo pathogens recovered in a randomised, controlled trial of impetigo treatment conducted in remote Indigenous communities of northern Australia.

**Methods:**

Each child had one or two sores, and the anterior nares, swabbed. All swabs were transported in skim milk tryptone glucose glycogen broth and frozen at –70°C, until plated on horse blood agar. *S. aureus* and *S. pyogenes* were confirmed with latex agglutination.

**Results:**

From 508 children, we collected 872 swabs of sores and 504 swabs from the anterior nares prior to commencement of antibiotic therapy. *S. pyogenes* and *S. aureus* were identified together in 503/872 (58%) of sores; with an additional 207/872 (24%) sores having *S. pyogenes* and 81/872 (9%) *S. aureus*, in isolation. Skin sore swabs taken during episodes with a concurrent diagnosis of scabies were more likely to culture *S. pyogenes* (OR 2.2, 95% CI 1.1 – 4.4, p = 0.03). Eighteen percent of children had nasal carriage of skin pathogens. There was no association between the presence of *S. aureus* in the nose and skin. Methicillin-resistance was detected in 15% of children who cultured *S. aureus* from either a sore or their nose. There was no association found between the severity of impetigo and the detection of a skin pathogen.

**Conclusions:**

*S. pyogenes* remains the principal pathogen in tropical impetigo; the relatively high contribution of *S. aureus* as a co-pathogen has also been confirmed. Children with scabies were more likely to have *S. pyogenes* detected. While clearance of *S. pyogenes* is the key determinant of treatment efficacy, co-infection with *S. aureus* warrants consideration of treatment options that are effective against both pathogens where impetigo is severe and prevalent.

**Trial registration:**

This trial is registered; ACTRN12609000858291.

**Electronic supplementary material:**

The online version of this article (doi:10.1186/s12879-014-0727-5) contains supplementary material, which is available to authorized users.

## Background

Impetigo is an epidermal infection caused by *Staphylococcus aureus* and *Streptococcus pyogenes*. It is common in Indigenous children of northern Australia, with prevalence as high as 70% [1]. The reported relative abundance of *S. aureus* and *S. pyogenes* has varied over time [2]. In recent decades, *S. aureus* and increasingly methicillin-resistant *S. aureus* (MRSA) has been the dominant reported pathogen in impetigo studies worldwide, most of which have taken place in temperate-climate regions, usually in affluent countries [3] where there is a low burden of disease. By contrast, in tropical regions, impetigo is far more common, and carries the greatest burden of sequelae [4]. *S. pyogenes* is assumed to have remained the dominant pathogen [5] but some reports are emerging on skin and soft tissue infections caused by *S. aureus* [6],[7]. There is limited microbiological surveillance of causative impetigo pathogens from high burden contexts and rates of antimicrobial resistance are often unknown [5]. Impetigo is strongly associated with scabies infestation in tropical environments [8], but the influence of scabies on the microbiology of impetigo has not previously been described. We report here on the microbiology of impetigo in a high burden setting, and explore the associations of this microbiology with age, sex, region, severity, presence of scabies and nasal carriage of skin pathogens. Our dataset derives from a large, non-inferiority randomised controlled trial (RCT) comparing trimethoprim-sulphamethoxazole (SXT) with benzathine penicillin G (BPG) for the treatment of impetigo in Indigenous children [9].

## Methods

### Study design

Indigenous children aged 3 months to 13 years were participants in the RCT. Children were eligible to participate on more than one occasion if at least 90 days had elapsed since their last involvement. As such, 508 children from 12 remote communities of the Northern Territory were enrolled for 663 episodes of impetigo; all analysis presented here has been restricted to a child’s first episode only. Six communities (comprising 463/508 children) were located in the tropical climatic region, commonly referred to as the “Top End”. The remaining communities were in Central Australia where a desert climate prevails. Children were stratified by impetigo severity. The severe stratum included children with ≥2 purulent or crusted sores and ≥5 overall body sores.

### Swabbing, transportation and culture methods

Each child had swabs taken from one or two sores (according to whether the episode was classified as mild or severe) prior to commencing antibiotics. A swab of the anterior nares was obtained to determine carriage of impetigo pathogens in the context of infection. Swabs were collected between 26 November 2009 and 20 November 2012. Rayon tipped cotton swabs (Copan, Italy) were transported at 4°C in 1 mL of skim milk tryptone glucose glycogen broth (STGGB) and frozen at −70°C within 5 days of collection. Swabs were defrosted, vortexed and an aliquot plated on horse blood agar and incubated for 48 hours at 37°C [10]. *S. aureus* and *S. pyogenes* were identified morphologically and confirmed with latex agglutination. All *S. aureus* isolates were staphytect (Oxoid, UK) and deoxyribonuclease (DNase, BD Diagnostics, USA) positive. *S. pyogenes* agglutinated with group A Lancefield antisera (Oxoid).

### Antimicrobial susceptibility testing

Antimicrobial susceptibility testing for *S. aureus* was determined on the Vitek2 platform using 22359 VITEK AST-P612 cards (bioMerieux, France) with Clinical and Laboratory and Standards Institute (CLSI, 2011) breakpoints utilised [11]. MRSA was defined as any *S. aureus* with a positive cefoxitin screen. Non-multidrug resistant MRSA (nmMRSA) was defined as MRSA resistant to <3 additional non beta-lactam antibiotics [12]. Multidrug-resistant MRSA (mMRSA) was defined as MRSA that was resistant to ≥3 non beta-lactam antibiotics [12].

We performed susceptibility testing for *S. pyogenes* with penicillin, erythromycin and clindamycin discs using CLSI disc diffusion standards. SXT susceptibility for *S. pyogenes* was determined with an E test® (bioMerieux) according to the European committee on antimicrobial susceptibility testing (EUCAST) standards (www.eucast.org, last accessed 15 November 2014). SXT susceptible strains had a MIC ≤1 mg/L and resistant isolates had a MIC >2 mg/L.

### Ethics statement

This study was approved by the Northern Territory Department of Health and Menzies School of Health Research Human Research Ethics Committee (09/08). Written informed consent was obtained from a child’s parent or guardian for all study procedures.

### Statistical analysis

Mixed-effects logistic regression (random effects accounting for correlated data due to multiple sores for the children in the severe impetigo strata) using Stata 13 (Statacorp, Texas, USA) was performed to assess associations between the growth of skin pathogens and the presence of scabies, severe impetigo, age, sex, region and nasal carriage.

## Results

### Baseline microbiology results

#### Sores

We obtained 872 swabs of sores from 508 children with untreated impetigo (median age 7 years, interquartile range [IQR] 5 – 9 years) at baseline. Seventy-two percent of children were in the severe impetigo stratum, all of whom had two sores swabbed. An impetigo pathogen was identified in 488/508 (96%) of children. From the 872 sores swabbed, *S. aureus* and *S. pyogenes* were identified together in 503/872 (58%), *S. pyogenes* alone in 207/872 (24%), and *S. aureus* alone in 81/872 (9%) of sores. Swabs from children in the severe impetigo stratum were not more likely to detect one or both skin pathogens compared with swabs from children in the mild impetigo stratum (Table 1). *S. aureus* was less likely to be detected in older children (OR 0.6, 95% CI 0.4 – 0.9 for children ≥ 5 years, Wald test on 2 degrees of freedom p = 0.04) or in children from Central Australia (OR 0.5, 95% CI 0.3 – 0.9, p = 0.02). Children from Central Australia were less likely to have sores co-infected with both *S. aureus* and *S. pyogenes* than children from the Top End (OR 0.5, 95% CI 0.3 – 0.9, p = 0.01).

**Table 1:** **Results from logistic regression models to assess associations between impetigo pathogens and age, sex, severity, presence of scabies and region**

Variable	*S. pyogenes* in sores		*S. aureus* in sores		Both *S. aureus and S. pyogenes* in sores		MRSA in sores positive for *S. aureus*	
	OR	95% CI	OR	95% CI	OR	95% CI	OR	95% CI
**Female**	1.3	0.8 – 2.1	1.0	0.7 – 1.4	1.1	0.8 – 1.5	1.0	0.6 – 1.7
**0-4 years**	1	(ref)	1	(ref)	1	(ref)	1	(ref)
**5-9 years**	1.1	0.7 – 2.0	0.6	0.4 – 0.9	0.8	0.5 – 1.1	0.9	0.5 – 1.6
**10 – 13 years**	1.2	0.6 – 2.4	0.5	0.3 – 0.9	0.7	0.4 – 1.2	0.7	0.3 – 1.5
**Severe strata**	1.4	0.8 – 2.6	0.7	0.4 – 1.1	1.1	0.7 – 1.7	0.6	0.3 – 1.1
**Scabies present**	2.2	1.1 – 4.4	0.8	0.5 – 1.3	0.9	0.6 – 1.4	1.4	0.7 – 2.6
**Central Australia**	1.2	0.5 – 2.8	0.5	0.3 – 0.9	0.5	0.3 – 0.9	1.1	0.4 – 2.7

#### Scabies

Scabies was diagnosed at baseline in 84/508 (17%) of children: by age group: 0-4 years (28/136, 21%); 5 – 9 years (40/271, 15%) and 10 – 13 years (16/101, 16%). Those with scabies present were more likely to also have *S. pyogenes* detected from sores (OR 2.2, 95% CI 1.1 – 4.4, p = 0.03) (Table 1).

#### Other beta haemolytic streptococci

Seven hundred and fifty-four beta haemolytic streptococci were cultured from skin and nose swabs of 508 children with impetigo. Of these, 740/754 (98%) were *S. pyogenes* with 710 from skin swabs and 30 from the nose swabs. The remaining beta haemolytic streptococci were group C (2 skin, 2 nose) and group G (7 skin, 3 nose).

### Antibiotic susceptibility

One isolate each of *S. pyogenes* and *S. aureus* were selected per child for reporting of the antibiotic susceptibility profile. Where possible, isolates cultured from skin sores were selected, with the remainder coming from swabs of the anterior nares.

#### S. pyogenes

There were 455 children with at least one *S. pyogenes* isolate available for antibiotic susceptibility assessment. All *S. pyogenes* isolates were susceptible to penicillin and erythromycin. Clindamycin resistance was detected in 9/455 (2%) *S. pyogenes* isolates. SXT resistance was detected in 4/455 (<1%) isolates at baseline with a MIC >2 mg/L.[13] The median SXT MIC for *S. pyogenes* was 0.094 (interquartile range, 0.094 – 0.125) mg/L.

#### S. aureus

There were 435 children with at least one *S. aureus* isolate available for antibiotic susceptibility assessment. Methicillin-resistance in *S. aureus* was detected in 65/435 (15%) isolates, all of which were nmMRSA. There was no detection of mMRSA. The respective resistance rates for other antibiotics by child were penicillin 413/435 (95%), SXT 3/435 (<1%), erythromycin 60/435 (14%) and fusidic acid 9/435 (2%). Inducible clindamycin resistance was reported in 60/435 (14%), 86% of which were MSSA. All 3 SXT resistant isolates were nmMRSA. Other antibiotics included on the VITEK card for *S. aureus* susceptibility testing all had resistance rates below 0.02%. For those with *S. aureus* detected, the presence of MRSA did not reach significance for sex, age group, severe strata, the presence of scabies or region (Table 1).

### Nasal carriage of skin pathogens

#### S. aureus

A nasal swab was taken at baseline for 504/508 (99%) of children. Before treatment, 91/504 (18%) children had confirmed carriage of a skin pathogen in the anterior nares. Both *S. aureus* and *S. pyogenes* were recovered from 16/91 (18%), *S. pyogenes* alone from 14/91 (15%) and *S. aureus* alone from 61/91 (67%). Of children with nasal carriage of *S. aureus*, 66/77 (86%) had MSSA and 11/77 (14%), MRSA. Nasal carriage of *S. aureus* and *S. pyogenes* was respectively 77/504 (15%) and 30/504 (6%).

We investigated the association of nasal carriage of *S. aureus* and the presence of *S. aureus* in impetigo lesions. There were 504 children with both skin and nose swabs available (Table 2). Surprisingly, of the 410 children culturing *S. aureus* on the skin, only 54 (13%) also harboured *S. aureus* in the nose. Based on the antibiogram, of the 54 with *S. aureus* in both the nose and skin sores, four had discordant *S. aureus* strains (i.e., MSSA at one site and MRSA at the other). Of the 94 children without *S. aureus* on the skin, there were 23 (24%) children who harboured *S. aureus* in the nose. Of 424 children harbouring *S. pyogenes* on the skin, 28 (7%) had concurrent growth of *S. pyogenes* from the anterior nares. There were 2 children with isolated growth of *S. pyogenes* from the anterior nares. Extending a model of Table 2, nasal colonisation with *S. aureus* was associated with less, not more, *S. aureus* on the skin (OR 0.6, 95% CI 0.4 – 1.0, p = 0.04), suggesting a separate epidemiology of *S. aureus* at these two sites.

**Table 2:** **Identification of** ***Staphylococcus aureus*** **from any impetigo lesion and the anterior nares for all children with at least one skin and nose swab available (n = 504 children)**

		Anterior nares		Total
		Positive	Negative	
**Impetigo**	**At least one sore positive**	54 (13%)	356 (87%)	410 (100%)
	**Negative**	23 (24%)	71 (76%)	94 (100%)
**Total**		**77 (15%)**	**427 (85%)**	**504** (100%)

## Discussion

*S. pyogenes* remains the key impetigo pathogen in Indigenous children of remote Australia. Co-infection with *S. aureus* is also highly prevalent. In addition, our study extends understanding of the microbiology of impetigo where scabies is endemic. Where scabies is present, we found that *S. pyogenes* is more likely to be recovered from impetigo lesions. We also found no positive correlation between nasal carriage of *S. aureus* and recovery of *S. aureus* from impetigo lesions, and no association between the severity of impetigo and recovery of either *S. pyogenes*, *S. aureus* or both.

The stronger association of *S. pyogenes* (than *S. aureus)* with scabies presence concurs with earlier work that linked scabies outbreaks with subsequent epidemics of post-streptococcal glomerulonephritis [14] and recommended treatment of scabies at a community level to reduce the complications of streptococcal pyoderma [15],[16]. Scabies association with impetigo in urban Indigenous children was similarly high to that found in our study at a rate of 25/111 (23%) [17]. No prior studies have reported on the association between scabies and MRSA detection. While the rates of both were high, we were unable to detect a significant association between these.

Both *S. pyogenes* and *S. aureus* have been reported as key impetigo pathogens, however the reported relative contributions of each have fluctuated over time and region. Previous microbiology studies of impetigo in both urban and remote Australian Indigenous children have shown high rates of co-infection [17],[18]. Valery *et al.* detected co-infection with skin pathogens in 54% of urban Indigenous children. Of those children who had both impetigo and scabies, *S. pyogenes* was recovered from 82% and *S. aureus* from 77% [17]. *S. pyogenes* remains an important skin pathogen in our region, as has also been reported from Fiji [8],[19] and supports the ongoing prescription of antibiotics active against *S. pyogenes* for the effective treatment of impetigo in tropical regions. Results of our clinical trial confirmed the importance of clearing *S. pyogenes* in order to achieve healing of impetigo lesions [9].

The isolated dominance of *S. aureus* and rising rates of MRSA reported elsewhere in skin and soft tissue infections [6],[20],[21] were not found in our study. Methicillin-resistance was detected in 15% of *S. aureus* isolates. This is lower than previously reported for our remote tropical context [22] and lower than recent rates found in a 20 year survey of *S. aureus* in our region [23] but consistent with a study of impetigo in urban Indigenous children of northern Australia [17]. We were uncertain whether MRSA varies by age, as this had not previously been explored, but we found no evidence that it does. The observation that MRSA rates were stable across age groups, suggests early colonisation of children in remote communities with MRSA strains.

We found that children in the severe strata, who had more than five purulent or crusted sores, were not more likely than children in the mild strata, to be infected with *S. pyogenes* and/or *S. aureus*. Although those stratified as mild, indicating fewer overall sores, may have had a less severe phenotype for each individual sore, the microbiology of these sores was no different. We thus provide robust evidence to refute earlier observational data in patients with impetigo where the association of co-infection with a more severe phenotype was suspected but not confirmed [24],[25].

Whilst throat carriage of *S. pyogenes* is commonly reported and is not thought to be the reservoir for skin infection, the anterior nares are thought to only rarely harbor *S. pyogenes* [26]. As such, throat swabs were not included in our study design as we focused on the characterization of nasal carriage of *S. aureus*. However, our findings of nasal carriage of *S. pyogenes* in 7% of children provide external validation of results from military recruits where nasal carriage of *S. pyogenes* was found in 8% of those with ecthyma [27]. There was no correlation between *S. aureus* nasal carriage and the recovery of *S. aureus* from impetigo lesions. Previous studies have concluded that when impetigo predominantly affects the lower limbs, there is either an absence of *S. aureus* nasal carriage or a different genotype [28]. Our data supports this observation in that 67% of impetigo episodes involved only the lower limbs [9]. As has previously been shown in military recruits [29], nasal decolonization is also unlikely to be a useful strategy in reducing the burden of impetigo in our tropical, endemic setting.

One study from Ghana in the 1970s with a similar tropical climate found impetigo to be dominated by Lancefield groups C and G streptococci [30]. These findings were not reproduced in our study and have not been confirmed in other published microbiology studies from our region [18] or other tropical contexts [31]. Non-group A streptococci do not appear to play a significant role in the pathogenesis of impetigo.

A strength of this study is the standardization of procedures for screening, swabbing and microbiological culture in the context of a clinical trial conducted according to International Conference on Harmonisation Good Clinical Practice guidelines. In addition, the large number of children recruited from 12 distinct communities from two regions of the Northern Territory suggests that the conclusions are rigorous. Genotypic assessment of the isolates was not conducted to determine the correlation of skin and nose isolates. The inability to correlate the molecular epidemiology with the phenotype is a limitation of this study. While the cost of molecular typing overall has become more affordable, the large number of isolates in this study made further molecular analysis cost prohibitive.

## Conclusions

*S. pyogenes* remains a key pathogen in impetigo in tropical contexts, despite the rise in *S. aureus* found in many industrialised and tropical settings. Our findings are in keeping with those reported from Fiji [8] and confirm that impetigo in tropical contexts continues to be driven by *S. pyogenes*. In the absence of local microbiology for impetigo, treatment algorithms should remain focussed on the treatment of *S. pyogenes*. However, we have demonstrated that co-infection with both *S. pyogenes* and *S. aureus* is likely. As such, consideration of treatment of impetigo with an agent that is effective against both *S. pyogenes* and *S. aureus* is important. We have also concluded that in the context of impetigo, there is no association between nasal colonisation and skin infection with *S. aureus*.

## Authors’ original submitted files for images

Below are the links to the authors’ original submitted files for images. Authors’ original file for figure 1 Authors’ original file for figure 2 Authors’ original file for figure 3 Authors’ original file for figure 4

## References

[1] Currie BJ, Carapetis JR (2000). Skin infections and infestations in Aboriginal communities in northern Australia. Australas J Dermatol.

[2] Koning S, van der Sande R, Verhagen AP, van Suijlekom-Smit LW, Morris AD, Butler CC, Berger M, van der Wouden JC (2012). Interventions for impetigo. Cochrane Database Syst Rev.

[3] Geria AN, Schwartz RA (2010). Impetigo update: new challenges in the era of methicillin resistance. Cutis.

[4] Carapetis JR, Steer AC, Mulholland EK, Weber M (2005). The global burden of group A streptococcal diseases. Lancet Infect Dis.

[5] Parks T, Smeesters PR, Steer AC (2012). Streptococcal skin infection and rheumatic heart disease. Curr Opin Infect Dis.

[6] Abdel Fattah NS, Darwish YW (2012). Antibiogram testing of pediatric skin infections in the era of methicillin-resistant Staphylococci aureus: an Egyptian University Hospital-based study. Int J Dermatol.

[7] Alvarez-Uria G, Reddy R (2012). Prevalence and antibiotic susceptibility of community-associated methicillin-resistant staphylococcus aureus in a rural area of india: is MRSA replacing methicillin-susceptible staphylococcus aureus in the community?. ISRN Dermatol.

[8] Steer AC, Jenney AW, Kado J, Batzloff MR, La Vincente S, Waqatakirewa L, Mulholland EK, Carapetis JR (2009). High burden of impetigo and scabies in a tropical country. PLoS Negl Trop Dis.

[9] Bowen AC, Tong SYC, Andrews RM, O'Meara IM, McDonald MI, Chatfield MD, Currie BJ, Carapetis JR: Short-course oral co-trimoxazole versus intramuscular benzathine benzylpenicillin for impetigo in a highly endemic region: an open-label, randomised, controlled, non-inferiority trial. *Lancet* 2014, 384(9960):2132–2140., 10.1016/S0140-6736(14)60841-2 25172376

[10] Bowen AC, Lilliebridge RA, Tong SY, Baird RW, Ward P, McDonald MI, Currie BJ, Carapetis JR (2012). Is streptococcus pyogenes resistant or susceptible to trimethoprim-sulfamethoxazole?. J Clin Microbiol.

[11] Svartman M, Finklea JF, Earle DP, Potter EV, Poon-King T (1972). Epidemic scabies and acute glomerulonephritis in Trinidad. Lancet.

[12] Taplin D, Porcelain SL, Meinking TL, Athey RL, Chen JA, Castillero PM, Sanchez R (1991). Community control of scabies: a model based on use of permethrin cream. Lancet.

[13] Carapetis JR, Connors C, Yarmirr D, Krause V, Currie BJ (1997). Success of a scabies control program in an Australian aboriginal community. Pediatr Infect Dis J.

[14] Valery PC, Wenitong M, Clements V, Sheel M, McMillan D, Stirling J, Sriprakash KS, Batzloff M, Vohra R, McCarthy JS (2008). Skin infections among Indigenous Australians in an urban setting in far North Queensland. Epidemiol Infect.

[15] McDonald MI, Towers RJ, Andrews RM, Benger N, Currie BJ, Carapetis JR (2006). Low rates of streptococcal pharyngitis and high rates of pyoderma in Australian aboriginal communities where acute rheumatic fever is hyperendemic. Clin Infect Dis.

[16] Jenney A, Holt D, Ritika R, Southwell P, Pravin S, Buadromo E, Carapetis J, Tong S, Steer A (2014). The clinical and molecular epidemiology of Staphylococcus aureus infections in Fiji. BMC Infect Dis.

[17] Iovino SM, Krantz KD, Blanco DM, Fernandez JA, Ocampo N, Najafi A, Memarzadeh B, Celeri C, Debabov D, Khosrovi B, Anderson M (2011). NVC-422 topical gel for the treatment of impetigo. Int J Clin Exp Pathol.

[18] Phakade RS, Nataraj G, Kuyare SS, Khopkar US, Mehta PR (2012). Is methicillin-resistant Staphylococcus aureus involved in community acquired skin and soft tissue infections? Experience from a tertiary care centre in Mumbai. J Postgrad Med.

[19] McDonald M, Dougall A, Holt D, Huygens F, Oppedisano F, Giffard PM, Inman-Bamber J, Stephens AJ, Towers R, Carapetis JR, Currie BJ (2006). Use of a single-nucleotide polymorphism genotyping system to demonstrate the unique epidemiology of methicillin-resistant Staphylococcus aureus in remote aboriginal communities. J Clin Microbiol.

[20] Tong SY, Varrone L, Chatfield MD, Beaman M, Giffard PM: Progressive increase in community-associated methicillin-resistant Staphylococcus aureus in Indigenous populations in northern Australia from 1993 to 2012. *Epidemiol Infect* 2014, 1–5., 10.1017/S0950268814002611 PMC9507213 25302939

[21] Barrow GI (1955). Clinical and bacteriological aspects of impetigo contagiosa. J Hyg (Lond).

[22] Hughes WT, Wan RT (1967). Impetigo contagiosa: etiology, complications, and comparison of therapeutic effectiveness of erythromycin and antibiotic ointment. Am J Dis Child.

[23] Masters PL, Brumfitt W, Mendez RL, Likar M (1958). Bacterial flora of the upper respiratory tract in Paddington families. 1952-4. Br Med J.

[24] Wasserzug O, Valinsky L, Klement E, Bar-Zeev Y, Davidovitch N, Orr N, Korenman Z, Kayouf R, Sela T, Ambar R, Derazne E, Dagan R, Zarka S (2009). A cluster of ecthyma outbreaks caused by a single clone of invasive and highly infective Streptococcus pyogenes. Clin Infect Dis.

[25] Barth JH (1987). Nasal carriage of staphylococci and streptococci. Int J Dermatol.

[26] Ellis MW, Griffith ME, Dooley DP, McLean JC, Jorgensen JH, Patterson JE, Davis KA, Hawley JS, Regules JA, Rivard RG, Gray PJ, Ceremuga JM, Sejoseph MA, Hospenthal DR (2007). Targeted intranasal mupirocin to prevent colonization and infection by community-associated methicillin-resistant Staphylococcus aureus strains in soldiers: a cluster randomized controlled trial. Antimicrob Agents Chemother.

[27] Belcher DW, Afoakwa SN, Osei-Tutu E, Wurapa FK, Osei L (1975). Letter: Non-group A streptococci in Ghanaian patients with pyoderma. Lancet.

[28] Lawrence DN, Facklam RR, Sottnek FO, Hancock GA, Neel JV, Salzano FM (1979). Epidemiologic studies among Amerindian populations of Amazonia. I. Pyoderma: prevalence and associated pathogens. Am J Trop Med Hyg.

[29] Bowen AC, Tong SY, Chatfield MD, Andrews RM, Carapetis JR (2013). Comparison of three methods for the recovery of skin pathogens from impetigo swabs collected in a remote community of Northern Territory, Australia. Trans R Soc Trop Med Hyg.

[30] Clinical and Laboratory Standards Institute (2014). Performance Standards for Antimicrobial Susceptibility Testing; Twenty-First Informational Supplement M100-S24.

[31] Tong SY, Bishop EJ, Lilliebridge RA, Cheng AC, Spasova-Penkova Z, Holt DC, Giffard PM, McDonald MI, Currie BJ, Boutlis CS (2009). Community-associated strains of methicillin-resistant Staphylococcus aureus and methicillin-susceptible S. aureus in indigenous Northern Australia: epidemiology and outcomes. J Infect Dis.

