# Supplementary material for: The microbiology of impetigo in Indigenous children: associations between Streptococcus pyogenes, Staphylococcus aureus,scabies, and nasal carriage
Source: BMC Infect Dis. 2014 Dec 31;14:727. doi: 10.1186/s12879-014-0727-5 (PMC4299569; doi:10.1186/s12879-014-0727-5)
Supplement: Supplementary file 1 — Authors’ original file for figure 1 [file 12879_2014_727_MOESM1_ESM.docx]

**Table 1:** Results from logistic regression models to assess associations between impetigo pathogens and age, sex, severity, presence of scabies and region.

| **Variable** | ***S. pyogenes* in sores** | | ***S. aureus* in sores** | | **Both *S. aureus and S. pyogenes* in sores** | | **MRSA in sores positive for *S. aureus*** | |
| --- | --- | --- | --- | --- | --- | --- | --- | --- |
|  | **OR** | **95% CI** | **OR** | **95% CI** | **OR** | **95% CI** | **OR** | **95% CI** |
| **Female** | 1.3 | 0.8 – 2.1 | 1.0 | 0.7 – 1.4 | 1.1 | 0.8 – 1.5 | 1.0 | 0.6 – 1.7 |
| **0-4 years** | 1 | (ref) | 1 | (ref) | 1 | (ref) | 1 | (ref) |
| **5-9 years** | 1.1 | 0.7 – 2.0 | 0.6 | 0.4 – 0.9 | 0.8 | 0.5 – 1.1 | 0.9 | 0.5 – 1.6 |
| **10 – 13 years** | 1.2 | 0.6 – 2.4 | 0.5 | 0.3 – 0.9 | 0.7 | 0.4 – 1.2 | 0.7 | 0.3 – 1.5 |
| **Severe strata** | 1.4 | 0.8 – 2.6 | 0.7 | 0.4 – 1.1 | 1.1 | 0.7 – 1.7 | 0.6 | 0.3 – 1.1 |
| **Scabies present** | 2.2 | 1.1 – 4.4 | 0.8 | 0.5 – 1.3 | 0.9 | 0.6 – 1.4 | 1.4 | 0.7 – 2.6 |
| **Central Australia** | 1.2 | 0.5 – 2.8 | 0.5 | 0.3 – 0.9 | 0.5 | 0.3 – 0.9 | 1.1 | 0.4 – 2.7 |
