# Supplementary material for: The microbiology of impetigo in Indigenous children: associations between Streptococcus pyogenes, Staphylococcus aureus,scabies, and nasal carriage
Source: BMC Infect Dis. 2014 Dec 31;14:727. doi: 10.1186/s12879-014-0727-5 (PMC4299569; doi:10.1186/s12879-014-0727-5)
Supplement: Supplementary file 2 — Authors’ original file for figure 2 [file 12879_2014_727_MOESM2_ESM.docx]

**Table2:** Identification of *Staphylococcus aureus* from any impetigo lesion and the anterior nares for all children with at least one skin and nose swab available (n=504 children)

|  | | **Anterior nares** | | **Total** |
| --- | --- | --- | --- | --- |
|  |  | **Positive** | **Negative** |  |
| **Impetigo** | **At least one sore positive** | 54 (13%) | 356 (87%) | 410 (100%) |
|  | **Negative** | 23 (24%) | 71 (76%) | 94 (100%) |
| **Total** | | **77 (15%)** | **427 (85%)** | **504** (100%) |
