# Supplementary material for: The microbiology of impetigo in Indigenous children: associations between Streptococcus pyogenes, Staphylococcus aureus,scabies, and nasal carriage
Source: BMC Infect Dis. 2014 Dec 31;14:727. doi: 10.1186/s12879-014-0727-5 (PMC4299569; doi:10.1186/s12879-014-0727-5)
Supplement: Supplementary file 3 — Authors’ original file for figure 3 [file 12879_2014_727_MOESM3_ESM.docx]

**Table 2:** Microbiology stratified by the presence of scabies

|  | **Scabies present (n=102)** | **Scabies absent (n=561)** | **P value** |
| --- | --- | --- | --- |
| No Growth | 1/102 (1.0%) | 25/561 (4.5%) | 0.096 |
| *S. aureus* and *S. pyogenes* | 78/102 (76.5%) | 417/561 (74.3%) | 0.648 |
| Total *S. pyogenes* | 97/102 (95.1%) | 496/561 (88.4%) | 0.043 |
| Total *S. aureus* | 82/102 (80.4%) | 457/561 (81.5%) | 0.799 |
