# Supplementary material for: The microbiology of impetigo in Indigenous children: associations between Streptococcus pyogenes, Staphylococcus aureus,scabies, and nasal carriage
Source: BMC Infect Dis. 2014 Dec 31;14:727. doi: 10.1186/s12879-014-0727-5 (PMC4299569; doi:10.1186/s12879-014-0727-5)
Supplement: Supplementary file 4 — Authors’ original file for figure 4 [file 12879_2014_727_MOESM4_ESM.docx]

**Table 3:** Identification of S*taphylococcus. aureus* from impetigo lesions and the anterior nares.

|  | | **Anterior nares** | | **Total** |
| --- | --- | --- | --- | --- |
|  |  | **Positive** | **Negative** |  |
| **Impetigo** | **Positive** | 81  (15.1%) | 457  (84.9%) | 538 |
|  | **Negative** | 28  (23.1%) | 93  (76.9%) | 121 |
| **Total** | | 109  (16.5%) | 550  (83.5%) | 659 |
